# Low Thoracic Muscle Mass: A Novel Indicator of Poor Prognosis in Patients With Severe Fever With Thrombocytopenia Syndrome

**DOI:** 10.1002/iid3.70271

**Published:** 2025-09-23

**Authors:** Xiao‐Min Wang, Pu Li, Lei Hu, Xiao Zhang, Jun Wang, Cheng‐Xin Zhu, Kun Lv, Da‐Wei Zhang

**Affiliations:** ^1^ Department of Radiology The First Affiliated Hospital of Anhui Medical University Hefei Anhui Province People's Republic of China; ^2^ Department of Vascular Surgery First Affiliated Hospital of Anhui Medical University Hefei Anhui Province People's Republic of China; ^3^ Department of Respiratory and Critical Care Medicine First Affiliated Hospital of Anhui Medical University Hefei Anhui Province People's Republic of China; ^4^ Department of Orthopedics Anhui Public Health Clinical Center Hefei Anhui Province People's Republic of China

**Keywords:** computed tomography, sarcopenia, SFTS, skeletal muscle index

## Abstract

**Background:**

Severe fever with thrombocytopenia syndrome (SFTS) poses a serious threat to public health due to its high mortality. Low thoracic muscle mass predicts poor clinical outcomes in many diseases, but its significance in SFTS remains unclear.

**Methods:**

This retrospective study involved 210 SFTS patients admitted to the First Affiliated Hospital of Anhui Medical University from 2020 to 2023. All subjects underwent chest computed tomography (CT) examination. Skeletal muscle index (SMI) was measured by CT images at the level of the twelfth thoracic vertebra (T12). The clinical data of patients from the survival and non‐survival groups were compared, and the prognostic value of T12‐SMI in SFTS patients was evaluated through least absolute shrinkage and selection operator regression and univariate and multivariate Cox regression.

**Results:**

Patients in the non‐survival group had a lower SMI. Receiver operating characteristic analysis indicated that SMI may predict adverse outcomes in patients with SFTS. Multivariate Cox regression analysis demonstrated that SMI was independently connected to negative outcomes of SFTS. T12‐SMI was correlated with albumin and creatinine.

**Conclusion:**

Low SMI is associated with adverse outcomes in patients with SFTS. SMI may serve as a reliable prognostic marker for SFTS in clinical settings.

## Graphical abstract

Low thoracic muscle mass is associated with adverse outcomes in patients with severe fever with thrombocytopenia syndrome. Thoracic muscle mass may serve as a reliable prognostic marker for severe fever with thrombocytopenia syndrome in clinical settings.

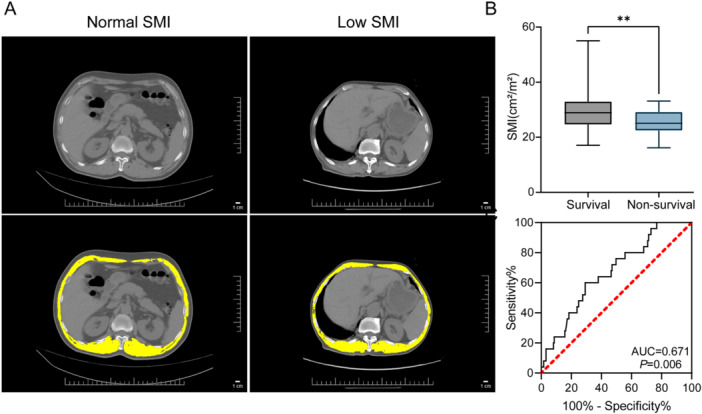

**Abbreviations**
APTT: activated partial thromboplastin time
BUN: blood urea nitrogen
CI: confidence interval
CREA: creatinine
CRP: c‐reactive protein
CT: computed tomography
CysC: cystatin C
DD: D‐dimer
EWGSOP: European Working Group on Sarcopenia in Older People
FDP: fibrinogen degradation products
HR: hazard ratio
KM: Kaplan–Meier
L3: 3 lumbar spines
LASSO: least absolute shrinkage and selection operator
PCT: procalcitonin
PLT: platelets
PNI: prognostic nutritional index
ROC: receiver operating characteristic
SD: standard deviation
SFTS: severe fever with thrombocytopenia syndrome
SMA: skeletal muscle area
SMI: skeletal muscle index
T12: twelfth thoracic vertebra
TT: thromboplastin time

## 1 Introduction

Severe fever with thrombocytopenia syndrome (SFTS) is an emerging viral infectious disease caused by a novel Bunyavirus [1]. SFTS is characterized by high morbidity and mortality, with a mortality rate of up to 6.4%–20.9% [2, 3], which has attracted increasing attention worldwide. In the early stages of SFTS, patients do not have typical clinical manifestations, and there may be challenges in the timely identification of high‐risk patients. The pathogenic process of SFTS is not well understood, and there are no specific therapies or effective vaccines [4]. Thus, it is critical to evaluate prognostic factors in patients with SFTS in the early time, which can be obtained on the day of admission, so that effective and targeted interventions can be taken in time to reduce the mortality of patients with SFTS.

The nutritional condition significantly impacts the progression of critically ill patients and is strongly linked to the outcomes of those with various diseases [5, 6, 7]. Reduced serum albumin, a conventional biomarker of nutritional status, is strongly linked to increased mortality in patients with SFTS [8]. In addition, the prognostic nutritional index (PNI) is a new index based on the total number of serum albumin and peripheral blood lymphocytes, which can assist in evaluating the immunonutritional status of patients [9]. Chunxia Guo found that SFTS patients exhibiting high C‐reactive protein (CRP)/PNI faced more severe illness and a higher risk of death than those with lower levels [10]. Nutritional status is difficult to be accurately quantified, and SFTS has a great impact on the coagulation function, liver and kidney function, and other laboratory indicators [11]. At present, the use of traditional serological indicators as markers of nutritional status assessment has certain limitations. According to the Global Leadership Initiative on Malnutrition [12], Low muscle mass is a phenotypic criterion for diagnosing malnutrition and is linked to poor clinical outcomes in critically ill patients [13, 14]. Therefore, the role of skeletal muscle mass in the progression of SFTS deserves further attention.

Sarcopenia is a condition marked by a decrease in skeletal muscle mass and strength for various reasons [15], which has gradually attracted attention in recent years. Low muscle mass is frequently linked with metabolic, physical, and functional issues, potentially impacting the outcomes of different diseases [16]. Recently, the European Working Group on Sarcopenia in Older People (EWGSOP) has identified computed tomography (CT) imaging as the premier method for measuring skeletal muscle mass in sarcopenia diagnosis [15, 17]. Specifically, the skeletal muscle index (SMI) at the third lumbar spine has been shown to be linked to the survival outcomes of patients with various infectious diseases [18]. However, since patients with SFTS often have pulmonary infection, chest CT is a routine examination for patients with SFTS. Studies have confirmed that there is a strong correlation between the 12th thoracic vertebrae (T12) and 3 lumbar spines (L3), which can also be used for the diagnosis of sarcopenia [19]. In patients with respiratory COVID‐19, low SMI at the thoracic T12 level measured by CT is connected to adverse outcomes [20]. Currently, there is no research investigating the association between sarcopenia and adverse outcomes in SFTS patients. Meanwhile, the clinical features and prognosis of SFTS patients with normal and low SMI were analyzed.

## 2 Methods

### 2.1 Subject

The study included SFTS patients admitted to the First Affiliated Hospital of Anhui Medical University between 2020 and 2023. The diagnosis of SFTS involves meeting one or more criteria: (1) SFTSV is isolated from the patient's blood; (2) SFTSV RNA is detected using real‐time reverse transcription polymerase chain reaction; (3) SFTSV virus‐specific IgM antibody is positive. The following were exclusion criteria: (1) incomplete data; (2) failure to perform a chest CT scan within 3 days of admission; (3) age < 18; (4) patients with Rickettsial, Dengue virus, Hantavirus and other acute virus infection; (5) patients with pregnancy, allergic diseases, autoimmune diseases, malignant tumors, kidney diseases; (6) patients who had used immunosuppressive drugs; (7) have a history of thoracic vertebra fractures or severe thoracic vertebra deformities; (8) imaging examinations suggest diseases of skeletal muscle status in patients with combined imaging: severe thoracic vertebral compression, spinal vascular malformations, malignant tumors, and so forth; (9) data that were not covered at the level of the T12 in chest CT scans; (10) inadequate CT image quality (e.g., presence of motion artifacts or spine implants). All patient clinical information and laboratory tests are collected from the patient's electronic medical record. All laboratory results for the study were from patients before treatment. The endpoint of the study was defined as death or discharge. All human experiments were approved by the ethical committee of First Affiliated Hospital of Anhui Medical University (NO. PJ2024‐12‐26).

### 2.2 Image Analysis

All CT scans were CT chest scans without IV contrast. The acquisition of CT images was accomplished by a radiology technician. SMI analysis was performed by two radiologists with more than 5 years of experience for the feature stability assay, who were also blinded to the information of the patients. Skeletal muscle area (SMA) was measured using semi‐automatic software sliceOmatic V5.0 (TomoVision, Magog, Canada). SMI is calculated by SMA/the square of height. We used Derstine et al. sex specific cut‐offs for low SMI in T12: for females, SMI < 20.8 cm^2^/m^2^; for males, SMI < 28.9 cm^2^/m^2^ [21]. Meanwhile, we also used the lowest gender specific quartile (male SMI < 27.54 cm^2^/m^2^, female SMI < 23.06 cm^2^/m^2^) as the cutoff value.

### 2.3 Statistical Analysis

SPSS 22.0 was used for data analysis in this study. Categorical variables are expressed as percentages (*n*, %) and tested using either a Chi‐square test, a continuous correction chi‐square test, or a Fisher's exact test. A *t*‐test is performed for continuous variables with a normal distribution and expressed as mean ± standard deviation (SD). The Mann–Whitney *U*‐test was performed for continuous variables with non‐normal distributions, expressed as median (interquartile range). Receiver operating characteristic (ROC) analysis was used to evaluate the diagnostic efficacy. Univariate Cox regression analysis was used to identify potential risk factors for SFTS. Parameters with *p* < 0.05 were included in subsequent analysis. To address the influence of multicollinearity between test measures, we used least absolute shrinkage and selection operator (LASSO) regression analysis to further screen for risk factors. Multivariate Cox regression analysis was used to identify the independent risk factors. The Kaplan–Meier (KM) method was used to assess 28‐day survival in cohorts classified by high or low SMI thresholds. Spearman correlation analysis was used to analyze the correlation between SMI and other test parameters. *p* < 0.05 was considered statistically significant.

## 3 Results

### 3.1 Demographics and Laboratory Parameters of Patients With SFTS

Two hundred and ten patients diagnosed with SFTS were included in the study, including 25 patients who died and 185 patients who survived. Table 1 displays the baseline clinical characteristics of patients who survived versus those who did not. Compared with the survival group, there were more patients over 65 years of age in the non‐survival group. The non‐survival group had a shorter hospital stay. There was no notable difference in disease history and comorbidities between the non‐survival and survival groups. In terms of clinical symptoms, the incidence of cough and neurological symptoms was higher in the non‐survival group. Laboratory tests revealed a drop in lymphocytes and platelets (PLT) in the non‐survival group. In addition, the levels of procalcitonin (PCT), blood urea nitrogen (BUN), creatinine (CREA), activated partial thromboplastin time (APTT), and thromboplastin time (TT), D‐dimer (DD), and fibrinogen degradation products (FDP) were increased in the non‐survival group (Table 2).

**Table 1:** Demographics of the survival and the non‐survival patients with SFTS.

Variables	Total (*n* = 210)	Survival (*n* = 185)	Non‐survival (*n* = 25)	*p*‐value
Age > 65 years, *n* (%)	107 (50.95)	88 (47.57)	19 (76.00)	0.008^b^
Male, *n* (%)	93 (44.29)	83 (44.86)	10 (40.00)	0.646^b^
Time from onset to admission (days)	5.00 (3.00, 7.00)	5.00 (3.50, 7.00)	5.00 (3.00, 6.50)	0.458^a^
Hospitalization > 14 days, *n* (%)	66 (31.43)	63 (34.05)	3 (12.00)	0.026^b^
Highest body temperature (°C)	39.00 (38.60, 39.40)	39.00 (38.60, 39.40)	39.00 (38.50, 39.70)	0.391^a^
Bite by ticks, *n* (%)	67 (31.90)	58 (31.35)	9 (36.00)	0.640^b^
History, *n* (%)				
Drink	23 (10.95)	20 (10.81)	3 (12.00)	1.000^d^
Smoke	25 (11.90)	23 (12.43)	2 (8.00)	0.754^d^
Diabetes	27 (12.86)	24 (12.97)	3 (12.00)	1.000^d^
Hypertensive disease	51 (24.29)	45 (24.32)	6 (24.00)	0.972^b^
CHD	6 (2.86)	4 (2.16)	2 (8.00)	0.315^d^
Cerebral infarction	19 (9.05)	16 (8.65)	3 (12.00)	0.860^d^
Liver disease	9 (4.29)	9 (4.86)	0 (0.00)	0.548^d^
Kidney disease	5 (2.38)	3 (1.62)	2 (8.00)	0.109^c^
Clinical manifestation, *n* (%)				
Shiver	91 (43.33)	84 (45.41)	7 (28.00)	0.099^b^
Fatigue	174 (82.86)	153 (82.70)	21 (84.00)	1.000^d^
Chest tightness	26 (12.38)	20 (10.81)	6 (24.00)	0.120^d^
Palpitation	8 (3.81)	8 (4.32)	0 (0.00)	0.615^d^
Muscular soreness	80 (38.10)	73 (39.46)	7 (28.00)	0.268^b^
Nausea	78 (37.14)	72 (38.92)	6 (24.00)	0.147^b^
Emesis	66 (31.43)	58 (31.35)	8 (32.00)	0.948^b^
Abdominal pain	32 (15.24)	29 (15.68)	3 (12.00)	0.854^d^
Diarrhea	112 (53.33)	99 (53.51)	13 (52.00)	0.887^b^
Pharyngalgia	7 (3.33)	7 (3.78)	0 (0.00)	0.692^d^
Cough	71 (33.81)	67 (36.22)	4 (16.00)	0.045^b^
Hemorrhage	45 (21.43)	38 (20.54)	7 (28.00)	0.394^b^
Neurological symptoms	40 (19.05)	31 (16.76)	9 (36.00)	0.043^d^
Signs, *n* (%)				
Skin change	19 (9.05)	19 (10.27)	0 (0.00)	0.191^d^
Hepatosplenomegaly	5 (2.38)	5 (2.70)	0 (0.00)	1.000^c^
Lymphadenopathy	51 (24.29)	46 (24.86)	5 (20.00)	0.594^b^

*Note:* Data are presented as numbers (%) or means (standard deviation) or median (interquartile range).

Abbreviations: CHD, coronary heart disease; SFTS, severe fever with thrombocytopenia syndrome.

^a^ Mann–Whitney *U*‐test.

^b^ *χ* ^2^ test.

^c^ Fisher test.

^d^ Continuous correction chi‐square test.

**Table 2:** Laboratory results of the survival and the non‐survival patients with SFTS.

Variables	Total (*n* = 210)	Survival (*n* = 185)	Non‐survival (*n* = 25)	*p*‐value
WBC (×10^9^/L)	2.44 (1.69, 4.23)	2.41 (1.70, 4.52)	2.76 (1.28)	0.678^b^
NEUT (×10^9^/L)	1.49 (1.01, 2.56)	1.41 (0.95, 2.61)	1.93 (0.94)	0.411^b^
LYMPH (×10^9^/L)	0.66 (0.44, 1.00)	0.67 (0.45, 1.04)	0.48 (0.39, 0.73)	0.047^b^
MONO (×10^9^/L)	0.14 (0.07, 0.29)	0.15 (0.07, 0.30)	0.12 (0.06, 0.19)	0.172^b^
PLT (×10^9^/L)	54.00 (37.50, 73.00)	56.00 (38.00, 74.75)	40.00 (29.00, 52.50)	0.015^b^
RBC (×10^12^/L)	4.33 (0.65)	4.35 (0.61)	4.17 (0.87)	0.190^a^
HGB (g/L)	130.25 (18.63)	130.71 (17.87)	126.80 (23.70)	0.325^a^
CRP (mg/L)	3.05 (0.93, 8.86)	2.99 (0.86, 8.31)	4.52 (2.23, 15.84)	0.088^b^
PCT (ng/mL)	0.16 (0.08, 0.35)	0.14 (0.08, 0.30)	0.37 (0.22, 0.83)	< 0.001^b^
CK (U/L)	408.00 (158.00, 925.00)	405.50 (158.00, 917.50)	539.00 (174.00, 968.00)	0.750^b^
LDH (U/L)	802.00 (509.00, 1324.00)	825.00 (530.00, 1285.00)	682.00 (401.50, 1827.25)	0.868^b^
ALT (U/L)	72.00 (45.50, 108.50)	73.00 (45.50, 105.00)	56.00 (41.00, 145.00)	0.941^b^
AST (U/L)	160.50 (93.00, 269.00)	163.00 (92.00, 264.00)	158.00 (97.50, 506.50)	0.458^b^
GGT (U/L)	33.00 (20.00, 68.00)	34.00 (20.00, 69.00)	29.00 (20.00, 63.00)	0.999^b^
ALB (g/L)	34.92 (5.85)	35.05 (6.00)	33.94 (4.57)	0.376^a^
GLO (g/L)	28.20 (25.38, 30.73)	28.10 (25.15, 30.65)	29.44 (4.63)	0.176^b^
ALP (U/L)	69.00 (58.00, 88.75)	70.00 (59.00, 91.50)	62.00 (53.00, 86.00)	0.261^b^
BUN (mmol/L)	5.79 (4.50, 7.73)	5.50 (4.40, 7.23)	8.20 (6.10, 10.07)	0.001^b^
CREA (μmol/L)	72.05 (59.00, 89.10)	69.20 (58.10, 86.55)	82.40 (66.15, 110.90)	0.010^b^
UA (μmol/L)	249.50 (192.50, 332.25)	247.00 (191.00, 330.00)	302.24 (140.26)	0.264^b^
PT (s)	13.10 (12.60, 13.80)	13.10 (12.58, 13.73)	13.64 (1.43)	0.354^b^
APTT (s)	49.20 (42.90, 58.90)	48.85 (42.30, 57.43)	54.90 (45.20, 68.15)	0.028^b^
TT (s)	21.50 (19.00, 28.30)	21.10 (18.90, 26.73)	27.00 (21.00, 45.25)	0.004^b^
DD (μg/mL)	2.71 (1.46, 5.75)	2.61 (1.41, 5.40)	4.18 (2.36, 8.53)	0.017^b^
FDP (mg/L)	8.39 (4.08, 19.49)	7.42 (3.94, 16.80)	15.79 (8.24, 32.68)	0.006^b^

*Note:* Data are presented as means (standard deviation) or median (interquartile range).

Abbreviations: ALB, albumin; ALP, alkaline phosphatase; ALT, alanine aminotransaminase; APTT, activated partial thromboplastin time; AST, aspartate aminotransferase; BUN, blood urea nitrogen; CK, creatine phosphokinase; CREA, creatinine; CRP, C‐reactive protein; DD, D‐dimer; FDP, fibrinogen degradation products; GGT, γ‐glutamyltransferase; GLO, globular protein; HGB, hemoglobin; LDH, lactate dehydrogenase; LYMPH, lymphocyte; MONO, monocyte; NEUT, neutrophil; PCT, procalcitonin; PLT, platelets; PT, prothrombin time; RBC, red blood cell; SFTS, severe fever with thrombocytopenia syndrome; TT, thromboplastin time; UA, uric acid; WBC, white blood cell.

^a^ *t*‐test.

^b^ Mann–Whitney *U*‐test.

### 3.2 Associations of T12‐SMI With Survival

**Figure 1:**
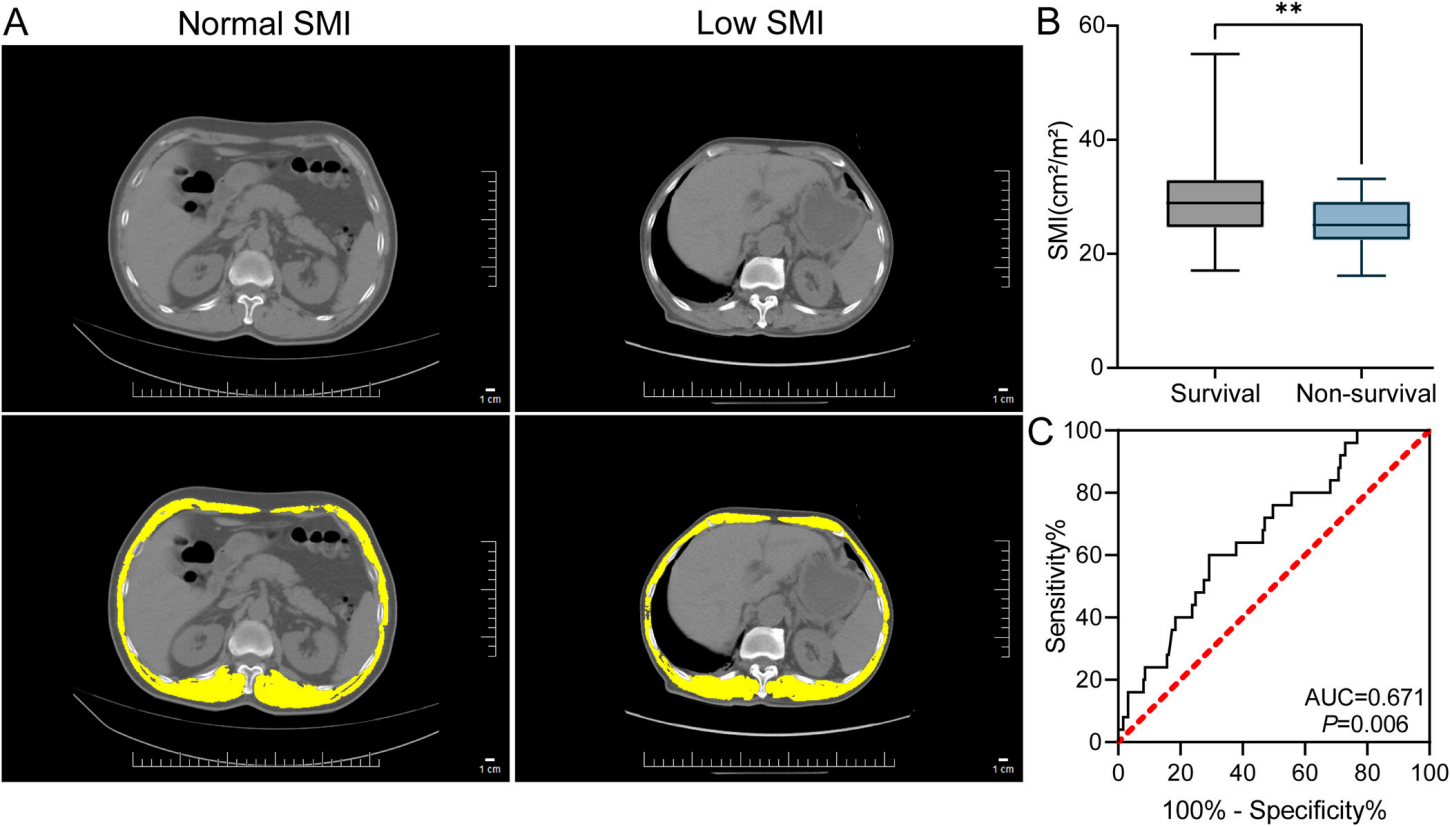
Comparison of skeletal muscle index in patients with SFTS. (A) Measurement of skeletal muscle area at the twelfth thoracic vertebra in patients with SFTS. (B) Comparison of skeletal muscle index in the survival and non‐survival groups. (C) ROC curve of SMI for the prognosis of SFTS patients. ROC, receiver operating characteristic; SFTS, severe fever with thrombocytopenia syndrome; SMI, skeletal muscle index. **p < 0.01.

As depicted in Figure 1A,B, the non‐survival group showed a decrease in thoracic skeletal muscle, with a significant drop in T12‐SMI. And ROC analysis showed that T12‐SMI had a certain predictive value in the poor prognosis of SFTS patients (AUC = 0.671; *p* = 0.006; Figure 1C).

### 3.3 T12‐SMI Is Independently Linked to Negative Outcomes in SFTS Patients

**Figure 2:**
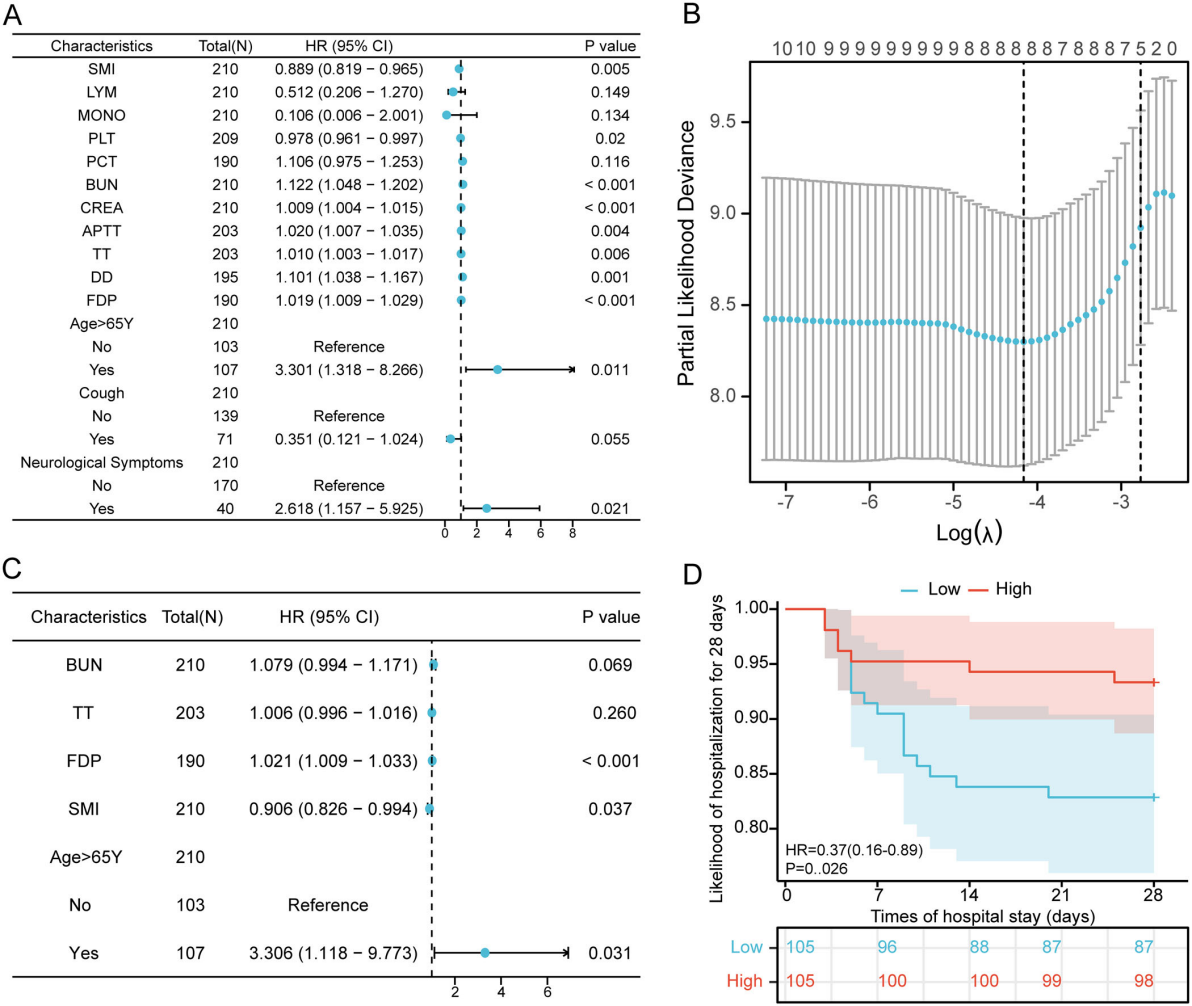
SMI is an independent risk factor for poor prognosis in patients with SFTS. (A) Univariate Cox regression analyses regarding the prognosis of patients with SFTS. (B) Selection of the optimal parameter (lambda) in the LASSO model for predicting the prognosis of patients with SFTS. (C) Multivariate Cox regression analyses regarding the prognosis of patients with SFTS. (D) KM survival curves according to the cutoff value of SMI. LASSO, least absolute shrinkage and selection operator; SFTS, severe fever with thrombocytopenia syndrome; SMI, skeletal muscle index.

Using univariate Cox regression analysis (Figure 2A), we identified that significant risk factors for SFTS include older age, neurological symptoms, lower T12‐SMI and PLT levels, and higher BUN, CREA, APTT, TT, FDP, and DD levels. To better reduce the problem of collinearity between variables, we included variables with *p* < 0.05 in univariate Cox regression into LASSO regression. SMI, BUN, TT, FDP, and age were further screened (Figure 2B). Then, a multivariate Cox regression model was performed. The results showed that SMI (hazard ratio [HR] = 0.906, 95% confidence interval [CI]: 0.826–0.994, *p* = 0.037), FDP (HR = 1.021, 95% CI: 1.009–1.033, *p* < 0.001), and age > 65 (HR = 3.306, 95%CI: 1.118–9.773, *p* < 0.001) were independent risk factors for death in SFTS patients (Figure 2C). Based on the results of Cox regression and LASSO regression, SMI is considered to be a key factor in the early prediction of adverse outcomes in SFTS patients.

### 3.4 Comparison Between Normal SMI and Low SMI Patients With SFTS

**Figure 3:**
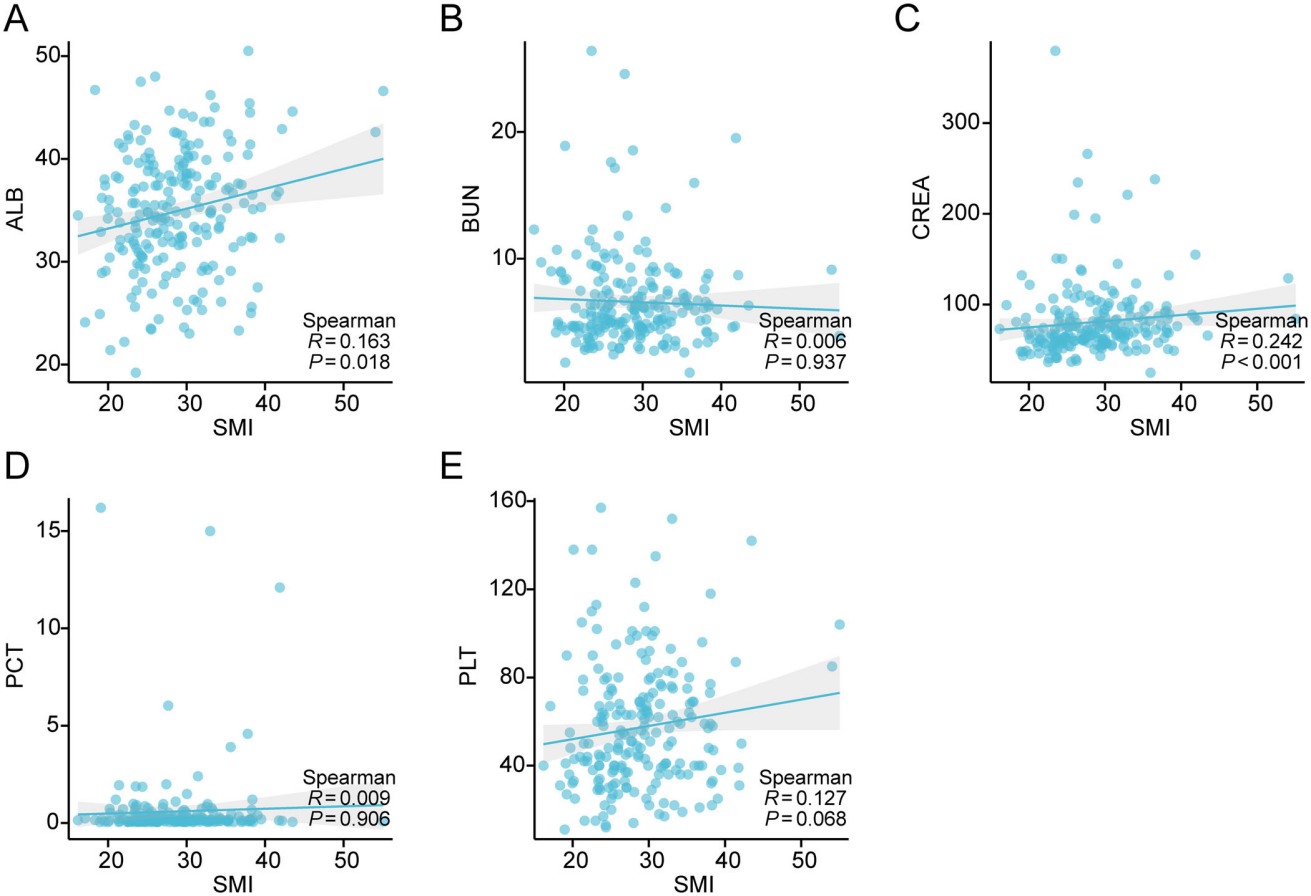
Correlation between SMI and laboratory tests in patients with SFTS. (A) The correlation analysis between SMI and ALB. (B) The correlation analysis between SMI and BUN. (C) The correlation analysis between SMI and CREA. (D) The correlation analysis between SMI and PCT. (E) The correlation analysis between SMI and PLT. ALB, albumin; BUN, blood urea nitrogen; CREA, creatinine; PCT, procalcitonin; PLT, platelets; SMI, skeletal muscle index.

We divided all patients into normal SMI and low SMI groups [21]. Figure 2D shows that the 28‐day survival rate for SFTS patients in the low SMI group was less than that of the normal SMI group, as determined by the KM method. Compared to those with normal SMI, individuals with low SMI tended to be older, predominantly male, and had a greater history of alcohol use. They reported fewer instances of nausea and vomiting, while neurological symptoms were more prevalent (Table 3). In addition, in terms of laboratory measures, patients with low SMI had higher BUN, CREA, and PCT. ALB and PLT were lower in patients with low SMI (Table 4). Considering that there is currently no accepted T12‐cutoff value for the diagnosis of sarcopenia, we also used the lowest gender specific quartile as the cutoff value, and then compared the data of patients with normal SMI and low SMI, and the results showed that the prognosis of patients with low SMI was still worse. Similarly, older age, a greater history of alcohol use, higher BUN, lower ALB, and PLT were also found in individuals with low SMI based on the lowest gender specific quartile as the cutoff value (Tables 5 and 6). Correlation analysis showed that SMI was positively correlated with ALB and CREA (Figure 3).

**Table 3:** Demographics of the normal and the low SMI patients with SFTS.

Variables	Normal SMI (*n* = 166)	Low SMI (*n* = 44)	*p*‐value
Age > 65 years, *n* (%)	75 (45.18)	32 (72.73)	0.001^b^
Male, *n* (%)	65 (39.16)	28 (63.64)	0.004^b^
Time from onset to admission (days)	5.00 (3.00, 7.00)	5.00 (3.00, 7.00)	0.982^a^
Hospitalization > 14 days, *n* (%)	56 (33.73)	10 (22.73)	0.162^b^
Highest body temperature (°C)	39.00 (38.70, 39.40)	39.00 (38.50, 39.30)	0.266^a^
Bite by ticks, *n* (%)	57 (34.34)	10 (22.73)	0.142^b^
History, *n* (%)			
Drink	13 (7.83)	10 (22.73)	0.011^d^
Smoke	18 (10.84)	7 (15.91)	0.356^b^
Diabetes	21 (12.65)	6 (13.64)	0.862^b^
Hypertensive disease	41 (24.70)	10 (22.73)	0.786^b^
CHD	4 (2.41)	2 (4.55)	0.608^c^
Cerebral infarction	14 (8.43)	5 (11.36)	0.759^d^
Liver disease	8 (4.82)	1 (2.27)	0.747^d^
Kidney disease	4 (2.41)	1 (2.27)	1.000^c^
Clinical manifestation, *n* (%)			
Shiver	76 (45.78)	15 (34.09)	0.164^b^
Fatigue	134 (80.72)	40 (90.91)	0.111^b^
Chest tightness	22 (13.25)	4 (9.09)	0.456^b^
Palpitation	8 (4.82)	0 (0.00)	0.297^d^
Muscular soreness	65 (39.16)	15 (34.09)	0.538^b^
Nausea	68 (40.96)	10 (22.73)	0.026^b^
Emesis	58 (34.94)	8 (18.18)	0.033^b^
Abdominal pain	26 (15.65)	6 (13.64)	0.740^b^
Diarrhea	90 (54.22)	22 (50.00)	0.618^b^
Pharyngalgia	6 (3.61)	1 (2.27)	1.000^d^
Cough	58 (34.94)	13 (29.55)	0.501^b^
Hemorrhage	35 (21.08)	10 (22.73)	0.813^b^
Neurological symptoms	27 (16.27)	13 (29.55)	0.046^b^
Signs, *n* (%)			
Skin change	16 (9.64)	3 (6.82)	0.776^d^
Hepatosplenomegaly	4 (2.41)	1 (2.27)	1.000^c^
Lymphadenopathy	43 (25.90)	8 (18.18)	0.288^b^

*Note:* Data are presented as numbers (%) or means (standard deviation) or median (interquartile range).

Abbreviations: CHD, coronary heart disease; SFTS, severe fever with thrombocytopenia syndrome.

^a^ Mann–Whitney *U*‐test.

^b^ *χ* ^2^ test.

^c^ Fisher test.

^d^ Continuous correction chi‐square test.

**Table 4:** Laboratory results of the normal and the low SMI patients with SFTS.

Variables	Normal SMI (*n* = 166)	Low SMI (*n* = 44)	*p*‐value
WBC (×10^9^/L)	2.41 (1.72, 4.23)	2.73 (1.60, 4.29)	0.914^b^
NEUT (×10^9^/L)	1.49 (1.02, 2.52)	1.52 (0.95, 2.57)	0.954^b^
LYMPH (×10^9^/L)	0.65 (0.45, 0.99)	0.73 (0.44, 1.14)	0.629^b^
MONO (×10^9^/L)	0.14 (0.07, 0.28)	0.13 (0.07, 0.31)	0.743^b^
PLT (×10^9^/L)	58.00 (40.00, 75.50)	40.00 (30.00, 54.00)	< 0.001^b^
RBC (×10^12^/L)	4.37 (0.60)	4.17 (0.78)	0.065^a^
HGB (g/L)	130.48 (17.88)	129.39 (21.45)	0.731^a^
CRP (mg/L)	3.16 (1.06, 8.68)	2.34 (0.50, 10.43)	0.283^b^
PCT (ng/mL)	0.14 (0.08, 0.31)	0.25 (0.14, 0.67)	0.007^b^
CK (U/L)	396.00 (148.00, 891.00)	522.50 (236.00, 991.00)	0.208^b^
LDH (U/L)	824.50 (506.75, 1274.75)	799.00 (509.00, 1733.00)	0.466^b^
ALT (U/L)	71.50 (45.50, 110.00)	74.00 (45.00, 93.00)	0.741^b^
AST (U/L)	154.00 (88.75, 264.00)	200.00 (101.25, 322.00)	0.131^b^
GGT (U/L)	34.00 (21.75, 66.25)	29.00 (17.25, 77.00)	0.505^b^
ALB (g/L)	35.46 (5.74)	32.87 (5.87)	0.009^a^
GLO (g/L)	28.10 (24.78, 30.73)	28.83 (4.48)	0.334^b^
ALP (U/L)	69.00 (58.00, 91.25)	69.50 (55.25, 83.25)	0.722^b^
BUN (mmol/L)	5.30 (4.38, 7.01)	8.35 (5.04, 10.38)	< 0.001^b^
CREA (μmol/L)	68.95 (58.00, 86.05)	76.90 (64.68, 105.60)	0.042^b^
UA (μmol/L)	247.00 (191.00, 323.50)	301.86 (139.14)	0.125^b^
PT (s)	13.10 (12.50, 13.70)	13.10 (12.70,14.48)	0.296^b^
APTT (s)	48.50 (42.40, 58.90)	52.35 (45.05, 63.80)	0.090^b^
TT (s)	21.20 (18.90, 27.00)	22.20 (19.55, 31.90)	0.118^b^
DD (μg/mL)	2.76 (1.46, 5.44)	2.48 (1.48, 7.29)	0.628^b^
FDP (mg/L)	8.22 (4.08, 17.57)	8.78 (4.17, 31.36)	0.361^b^

*Note:* Data are presented as means (standard deviation) or median (interquartile range).

Abbreviations: ALB, albumin; ALP, alkaline phosphatase; ALT, alanine aminotransaminase; APTT, activated partial thromboplastin time; AST, aspartate aminotransferase; BUN, blood urea nitrogen; CK, creatine phosphokinase; CREA, creatinine; CRP, C‐reactive protein; DD, D‐dimer; FDP, fibrinogen degradation products; GGT, γ‐glutamyltransferase; GLO, globular protein; HGB, hemoglobin; LDH, lactate dehydrogenase; LYMPH, lymphocyte; MONO, monocyte; NEUT, neutrophil; PCT, procalcitonin; PLT, platelets; PT, prothrombin time; RBC, red blood cell; SFTS, severe fever with thrombocytopenia syndrome; TT, thromboplastin time; UA, uric acid; WBC, white blood cell.

^a^ *t*‐test.

^b^ Mann–Whitney *U*‐test.

**Table 5:** Demographics of the normal and the low SMI patients with SFTS according to the lowest gender specific quartile.

Variables	Normal SMI (*n* = 158)	Low SMI (*n* = 52)	*p*‐value
Age > 65 years, *n* (%)	69 (43.67)	38 (73.08)	< 0.001^b^
Male, *n* (%)	70 (44.30)	23 (44.23)	0.993^b^
Time from onset to admission (days)	5.00 (3.00,7.00)	5.00 (3.25,7.00)	0.363^a^
Hospitalization > 14 days, *n* (%)	53 (33.54)	13 (25.00)	0.250^b^
Highest body temperature (°C)	39.00 (38.70,39.50)	39.00 (38.50,39.23)	0.114^a^
Bite by ticks, *n* (%)	57 (36.08)	10 (19.23)	0.024^b^
History, *n* (%)			
Drink	13 (8.23)	10 (19.23)	0.028^c^
Smoke	18 (11.39)	7 (13.46)	0.689^b^
Diabetes	23 (14.56)	4 (7.69)	0.200^b^
Hypertensive disease	42 (26.58)	9 (17.31)	0.176^b^
CHD	4 (2.53)	2 (3.85)	0.639^c^
Cerebral infarction	13 (8.23)	6 (11.54)	0.658^d^
Liver disease	7 (4.43)	2 (3.85)	1.000^d^
Kidney disease	4 (2.53)	1 (1.92)	1.000^c^
Clinical manifestation, *n* (%)			
Shiver	74 (46.84)	17 (32.69)	0.074^b^
Fatigue	125 (79.11)	49 (94.23)	0.012^b^
Chest tightness	21 (13.29)	5 (9.62)	0.485^b^
Palpitation	8 (5.06)	0 (0.00)	0.216^d^
Muscular soreness	61 (38.61)	19 (36.54)	0.790^b^
Nausea	59 (37.34)	19 (36.54)	0.917^b^
Emesis	50 (31.65)	16 (30.77)	0.906^b^
Abdominal pain	22 (13.92)	10 (19.23)	0.356^b^
Diarrhea	86 (54.43)	26 (50.00)	0.579^b^
Pharyngalgia	6 (3.80)	1 (1.92)	0.835^d^
Cough	56 (35.44)	15 (28.85)	0.383^b^
Hemorrhage	34 (21.52)	11 (21.15)	0.956^b^
Neurological symptoms	26 (16.46)	14 (26.92)	0.095^b^
Signs, *n* (%)			
Skin change	17 (10.76)	2 (3.85)	0.219^d^
Hepatosplenomegaly	4 (2.53)	1 (1.92)	1.000^c^
Lymphadenopathy	41 (25.95)	10 (19.23)	0.327^b^
Mortality, *n* (%)	13 (8.23)	12 (23.08)	0.004^b^

*Note:* Data are presented as numbers (%) or means (standard deviation) or median (interquartile range).

Abbreviations: CHD, coronary heart disease; SFTS, severe fever with thrombocytopenia syndrome.

^a^ Mann–Whitney *U*‐test.

^b^ *χ* ^2^ test.

^c^ Fisher test.

^d^ Continuous correction chi‐square test.

**Table 6:** Laboratory results of the normal and the low SMI patients with SFTS according to the lowest gender specific quartile.

Variables	Normal SMI (*n* = 158)	Low SMI (*n* = 52)	*p*‐value
WBC (×10^9^/L)	2.50 (1.71, 4.51)	2.31 (1.66, 3.91)	0.556^b^
NEUT (×10^9^/L)	1.58 (1.04, 2.76)	1.30 (0.95, 2.26)	0.244^b^
LYMPH (×10^9^/L)	0.64 (0.44, 1.00)	0.75 (0.45, 1.05)	0.212^b^
MONO (×10^9^/L)	0.14 (0.07, 0.29)	0.15 (0.09, 0.29)	0.379^b^
PLT (×10^9^/L)	58.00 (39.50, 75.00)	41.50 (32.25, 59.00)	0.005^b^
RBC (×10^12^/L)	4.39 (0.66)	4.17 (0.59)	0.035^a^
HGB (g/L)	130.90 (19.29)	128.27 (16.49)	0.379^a^
CRP (mg/L)	3.01 (1.01, 8.68)	3.12 (0.50, 11.48)	0.783^b^
PCT (ng/mL)	0.14 (0.08, 0.31)	0.22 (0.09, 0.59)	0.102^b^
CK (U/L)	408.00 (148.00,923.00)	420.00 (198.25, 927.50)	0.690^b^
LDH (U/L)	854.00 (513.00, 1317.25)	796.00 (490.00, 1545.00)	0.973^b^
ALT (U/L)	71.50 (43.75,113.00)	73.00 (49.00,1545.00)	0.801^b^
AST (U/L)	146.50 (88.75, 264.75)	202.00 (112.00,310.00)	0.059^b^
GGT (U/L)	34.50 (20.75, 68.00)	28.50 (19.25, 69.25)	0.547^b^
ALB (g/L)	35.47 (5.67)	33.22 (6.10)	0.016^a^
GLO (g/L)	28.00 (24.70, 30.73)	28.55 (26.35, 30.78)	0.181^b^
ALP (μ/L)	69.00 (58.00, 88.00)	70.50 (57.75, 93.65)	0.572^b^
BUN (mmol/L)	5.65 (4.40, 7.19)	6.00 (4.67, 9.88)	0.043^b^
CREA (μmol/L)	72.05 (60.00, 87.08)	70.50 (58.05, 93.65)	0.822^b^
UA (μmol/L)	252.50 (197.00, 325.50)	242.00 (164.50, 363.00)	0.814^b^
PT (s)	13.10 (12.60, 13.90)	13.14 (1.07)	0.251^b^
APTT (s)	49.30 (43.20, 59.30)	48.20 (41.83, 58.53)	0.745^b^
TT (s)	21.20 (19.00, 26.80)	22.15 (19.35, 33.08)	0.186^b^
DD (μg/mL)	2.71 (1.57, 5.75)	2.55 (1.41, 5.72)	0.874^b^
FDP (mg/L)	8.13 (4.14, 19.02)	8.95 (3.92, 21.00)	0.759^b^

*Note:* Data are presented as means (standard deviation) or median (interquartile range).

Abbreviations: ALB, albumin; ALP, alkaline phosphatase; ALT, alanine aminotransaminase; APTT, activated partial thromboplastin time; AST, aspartate aminotransferase; BUN, blood urea nitrogen; CK, creatine phosphokinase; CREA, creatinine; CRP, C‐reactive protein; DD, D‐dimer; FDP, fibrinogen degradation products; GGT, γ‐glutamyltransferase; GLO, globular protein; HGB, hemoglobin; LDH, lactate dehydrogenase; LYMPH, lymphocyte; MONO, monocyte; NEUT, neutrophil; PCT, procalcitonin; PLT, platelets; PT, prothrombin time; RBC, red blood cell; SFTS, severe fever with thrombocytopenia syndrome; TT, thromboplastin time; UA, uric acid; WBC, white blood cell.

^a^ *t*‐test.

^b^ Mann–Whitney *U*‐test.

## 4 Discussion

A major challenge in improving the ability to treat patients with SFTS is the early identification of patients with poor prognosis. Thus, identifying convenient, effective, and cost‐efficient indicators to forecast the mortality outcome of SFTS is essential. In recent years, some studies have described the correlation between death outcome and various laboratory indicators [22, 23, 24], but no studies have been conducted on the relationship between imaging indicators and patient prognosis. The purpose of this study was to explore the link between T12‐SMI and negative outcomes in patients with SFTS. This single‐center retrospective study suggests that T12‐SMI is an independent risk factor for death in patients with fever with thrombocytopenia syndrome. Individuals in the sarcopenia group faced a greater risk of mortality and were in poorer health compared to those without sarcopenia. Through early attention to skeletal muscle and nutritional status of patients, critical patients can be identified in time, and comprehensive treatment strategies can be developed to ultimately improve the prognosis of SFTS patients.

We first compared clinical information and laboratory indicators for surviving and dying SFTS patients. Consistent with previous research, our study suggests that SFTS deaths occur at an older age. In addition, we found a significant increase in the incidence of cough and neurological symptoms in the deceased group. In recent years, studies have found that SFTS patients are prone to pulmonary infection, and SFTS patients with lung infections have a higher mortality rate compared to those without co‐infections [25]. Previous studies have shown that SFTS patients with respiratory symptoms, bleeding manifestations, exudation symptoms, nervous system symptoms, and organ failure are more likely to be co‐infected [26]. A study on SFTS combined with invasive pulmonary aspergillus suggests that diabetes, cough, and neurological symptoms are risk factors for invasive pulmonary aspergillus [25]. Another study on SFTS showed that 32% of patients had respiratory symptoms and even respiratory failure, and all of these patients were severe cases and eventually ended in death [27]. Numerous studies have discovered a strong link between neurological symptoms and the mortality rate of SFTS patients [28, 29]. The study found that hemophagocytic cells were found in the cerebrospinal fluid of SFTS patients, indicating that inflammation storms might indirectly cause consciousness disturbances in SFTS patients [30, 31]. Serum cytokines and viral load are closely related to central nervous system involvement in SFTS patients. Therefore, for SFTS patients with clinical manifestations of expectoration and central nervous system symptoms, it is important to be aware of the risk of lung infections and provide early treatment.

In terms of laboratory indicators, we found that the lymphocytes and PLT in the non‐survival group were decreased, and the inflammatory indicators PCT, renal function indicators BUN and CREA, and coagulation indicators APTT, TT, DD, and FDP were significantly increased. Studies have shown that the mechanism of thrombocytopenia and leukopenia in patients with SFTS is currently considered to be caused by cytokine storm and immune dysfunction after SFTSV infection. SFTSV infection can damage T lymphocytes through the Fas/FasL‐mediated apoptosis pathway, and the SFTSV also targets immature B cells that express PAX5, possibly leading to severe lymphopenia [32, 33]. The kidney is also one of the most important target organs of SFTSV infection. BUN and CREA reflect kidney damage, and in SFTS patients, BUN and CREA levels varied significantly between the non‐survival and survival groups. Severe SFTS patients are often accompanied by acute kidney injury, but the mechanism of kidney injury in patients with SFTS is still unclear. SFTSV viral load in the kidney and urine of SFTS patients is significantly increased [34], so SFTSV may directly damage the kidney. Interestingly, lymphocyte count and CREA are also commonly used to assess nutritional status. Malnutrition is common among middle‐aged and older adults and can impact patient clinical outcomes. In critically ill patients, malnutrition is associated with poor clinical outcomes. There are many patients with severe SFTS. The nutritional status may be related to the prognosis of SFTS.

Low levels of skeletal muscle mass are significant markers of the body's nutritional condition [35], which is an independent risk factor for patients with liver cirrhosis and cancer [36, 37]. The skeletal muscle density and area play a key role in evaluating the prognosis of critically ill patients [38]. There are many different modalities for assessing muscle mass in the body, including bioelectrical impedance analysis, dual energy X‐ray absorptiometry, magnetic resonance imaging, and CT, with CT being increasingly used to assess muscle mass [17]. In this study, we used T12‐SMI to assess skeletal muscle mass in patients with SFTS. The results showed that T12‐SMI decreased significantly in the non‐survival group, and SMI had a certain predictive effect on the death of patients. The analyses using univariate Cox regression, LASSO regression, and multivariate Cox regression revealed that T12‐SMI independently predicts the risk of death in SFTS patients. At present, there are few studies on SMI and viral diseases. The latest research shows that T12‐SMI is a risk factor for acquired infection, increased length of hospital stays, and ICU admission in COVID‐19 patients [20]. Therefore, low skeletal muscle mass deserves more attention due to its strong prognostic value in predicting mortality in patients with SFTS.

At present, there is a lack of recommended diagnostic criteria for sarcopenia based on CT, and there are different cut‐off values of SMI in previous studies. Derstine's criteria and the lowest gender specific quartile were used for grouping in this study [21]. Upon analyzing the demographic data, we discovered that SFTS patients with sarcopenia tended to be older, predominantly male, and exhibited higher rates of alcohol use, nausea, and vomiting. Studies have shown that about 5%–10% of people over 65 years old in China have sarcopenia, and people over 50 years old have 1%–2% muscle loss every year [39]. The reason for the higher prevalence of SFTS with sarcopenia found in men than in women in this study may be that men have higher rates of smoking and drinking, and smoking reduces protein synthesis and accelerates protein degradation, leading to sarcopenia [40]. At the same time, the results of this investigation show that drinking history is also a risk factor for patients with sarcopenia, which may be because long‐term alcohol intake leads to muscle type Ⅱ fiber atrophy, resulting in muscle mass reduction and sarcopenia [41]. Insufficient intake is also the main cause of sarcopenia. The normal living body needs a variety of vital nutrients to participate in the survival state, but in SFTS patients, the nutritional intake is not up to standard. In clinical practice, patients often have symptoms of anorexia, nausea, and vomiting, and food intake is significantly reduced, so they are more likely to develop sarcopenia.

In the comparison of laboratory indexes, we found that SFTS patients with sarcopenia had lower PLT and albumin, and higher PCT, BUN, and CREA. The changes in these indices suggested that SFTS patients with sarcopenia were more critical. SMI was found to be associated with albumin and CREA according to the correlation analysis. Viewed as a key marker of nutritional health, albumin might contribute synergistically to the heightened risk of adverse events associated with sarcopenia [42]. Serum albumin is a predictor of morbidity, mortality, and prolonged hospital stay in critically ill patients [43]. When the body is infected with SFTSV, the catabolism of albumin is increased, and is often accompanied by liver and kidney function damage, resulting in impaired liver synthesis function and decreased albumin level. CREA is a product of muscle metabolism and, to a certain extent, serum CREA levels reflect endogenous muscle mass [44]. When renal function status is stable, CREA production is relatively constant and thus can be used to assess skeletal muscle mass [45]. Tabara et al. validated that serum CREA/Cystatin C (CysC) was significantly associated with decreased skeletal muscle strength and mass in a community elderly population [46]. Hirai et al. [47] proposed that patients with a lower CREA/CysC ratio are at high risk of acute severe exacerbation of chronic obstructive pulmonary disease. SFTS is not suitable for patients with SFTS because of its great damage to renal function. CT can more accurately evaluate skeletal muscle mass in patients with SFTS.

This study is the initial one to assess the prognosis of skeletal muscle mass in SFTS patients using CT, which is of great significance for the inclusion of skeletal muscle in the treatment and prognosis evaluation of SFTS patients in the future, but there are still some shortcomings in this study. First, this study was a single‐center study with few case data, and data from a larger sample are needed to support our results in the future. Second, there is a lack of dynamic monitoring and observation of skeletal muscle mass and function, as well as long‐term survival follow‐up data of SFTS patients. Additional research is required to assess the long‐term prognostic impact of sarcopenia in patients with SFTS.

## 5 Conclusion

Low skeletal muscle mass has been identified as an independent risk factor for poor outcomes in patients with SFTS. The T12‐SMI index serves as a valuable tool for risk stratification and management of these patients, with individuals exhibiting sarcopenia being classified as high‐risk. Evaluating skeletal muscle mass not only helps predict mortality in SFTS patients but also aids in tailoring more precise therapeutic interventions.

## Author Contributions

**Xiao‐Min Wang:** conceptualization, validation, formal analysis, investigation, writing – original draft. **Pu Li:** data curation, investigation, methodology. **Lei Hu:** formal analysis, investigation, software. **Xiao Zhang:** investigation, supervision. **Jun Wang:** investigation, visualization. **Cheng‐Xin Zhu:** investigation. **Kun Lv:** investigation. **Da‐Wei Zhang:** conceptualization, funding acquisition, writing – review and editing.

## Ethics Statement

The ethics in this study were approved by the ethics committee of First Affiliated Hospital of Anhui Medical University (NO. PJ2024‐12‐26). The study was conducted retrospectively; there was no need to seek informed consent from each patient.

## Conflicts of Interest

The authors declare no conflicts of interest.

## Data Availability

The data that support the findings of this study are available on request from the corresponding author. There were no publicly available data sets in this study. The data that support the findings of this study are available on request from the corresponding author. The data are not publicly available due to privacy or ethical restrictions.
